# Paragonimiasis pulmonar crónica en niño indígena kichwa contagiado en la amazonia ecuatoriana

**DOI:** 10.7705/biomedica.7469

**Published:** 2025-03-28

**Authors:** Manuel Calvopina, Elías David Guaman-Charco, Jeremmy Erazo-Coello, Verónica Osorio-Pozo, Angelita Cabrera-Aguilar, Mariella Anselmi

**Affiliations:** 1 One Health Research Group, Facultad de Medicina, Universidad de las Américas, Quito, Ecuador Universidad de las Américas Universidad de las Américas Quito Ecuador; 2 Hospital General Marco Vinicio Iza, Ministerio de Salud Pública, Nueva Loja, Sucumbíos, Ecuador Hospital General Marco Vinicio Iza Hospital General Marco Vinicio Iza Ministerio de Salud Pública Nueva Loja Sucumbíos Ecuador; 3 Servicio de Neumología, Hospital Pediátrico Baca Ortiz, Ministerio de Salud Pública, Quito, Ecuador Hospital Pediátrico Baca Ortiz Hospital Pediátrico Baca Ortiz Ministerio de Salud Pública Quito Ecuador; 4 Facultad de Ciencias Médicas, Universidad Central del Ecuador, Quito, Ecuador Universidad Central del Ecuador Universidad Central del Ecuador Quito Ecuador; 5 Centro de Epidemiología Comunitaria y Medicina Tropical, Esmeraldas, Ecuador Centro de Epidemiología Comunitaria y Medicina Tropical Centro de Epidemiología Comunitaria y Medicina Tropical Esmeraldas Ecuador

**Keywords:** paragonimiasis, pediatría, ecosistema amazónico, Ecuador, Paragonimiasis, paediatrics, Amazonian ecosystem, Ecuador

## Abstract

La paragonimiasis es una parasitosis causada por el trematodo *Paragonimus spp*. La Organización Mundial de la Salud la considera una enfermedad tropical desatendida, clasificada como alimentaria, causada por la ingestión de crustáceos de agua dulce, infectados, crudos o insuficientemente cocidos. En Ecuador, es endémica en las regiones tropicales de la de la costa y la amazonia.

Se presenta el caso de un niño kichwa de diez años, originario de una comunidad rural de la región amazónica, diagnosticado en el Hospital Pediátrico de Quito. El niño presentaba síntomas de tos y expectoración herrumbrosa desde hacía cuatro años, con antecedentes de ingestión de cangrejos. En la tomografía computarizada se observaron cambios en el parénquima pulmonar sugestivos de paragonimiasis pulmonar. El diagnóstico se confirmó mediante la observación microscópica de huevos operculados de *Paragonimus spp*. en el esputo. Se administró triclabendazol durante dos días y los controles posteriores mostraron resultados negativos en los esputos.

Se discute la posibilidad del diagnóstico en regiones no endémicas, así como la falta de sospecha clínica y del diagnóstico con pruebas de laboratorio en las zonas endémicas. Además, se señala la carencia de los fármacos de elección -triclabendazol y praziquantel- en Ecuador.

## Abstract (en)

Paragonimiasis is caused by the trematode *Paragonimus spp*. and is considered a foodborne trematodiasis; it is classified as a neglected tropical disease by the World Health Organization, primarily transmitted through the consumption of infected and undercooked freshwater crustaceans. In Ecuador, it predominantly affects tropical regions such as the Coast and the Amazon.

We present the case of a ten-year-old Kichwa boy from a rural Amazonian community, diagnosed at the Hospital Pediátrico de Quito. The child presented persistent cough and rusty sputum for four years, with a history of eating crabs. Computed tomography indicated pulmonary parenchymal alterations. The diagnosis of pulmonary paragonimiasis was confirmed via microscopic identification of operculated *Paragonimus spp*. eggs in the sputum. Treatment with triclabendazole for two days resulted in subsequent negative sputum findings during follow-up examinations.

We discuss the possibility of diagnosis in non-endemic regions and the lack of clinical suspicion and laboratory diagnosis in endemic areas. Furthermore, we highlight the shortage of the drugs of choice, triclabendazole, and praziquantel, in Ecuador.

La paragonimiasis es una enfermedad zoonótica causada por el trematodo del género *Paragonimus*, presente en países asiáticos, africanos y americanos, incluyendo Ecuador, Colombia, Brasil, Perú y los Estados Unidos, entre otros ^1^^,^^2^. En Ecuador, esta parasitosis es endémica en las regiones tropicales y subtropicales de la costa y la amazonia; *Paragonimus mexicanus* es una de las especies infecciosas ^3^^,^^4^. El primer caso fue reportado en 1922 ^5^ y, según Amunarriz, en la amazonia ecuatoriana, esta enfermedad es frecuente entre los indígenas kichwa y shuar, habitantes de las orillas del río Napo ^6^. Los estudios de captación activa en estas comunidades reportaron una prevalencia del 51,2 % ^7^.

La Organización Mundial de la Salud (OMS) clasifica la paragonimiasis como una enfermedad tropical desatendida y la incluye entre las trematodiasis transmitidas por alimentos ^1^. La infección humana ocurre por consumir crustáceos de río, infectados, crudos o insuficientemente cocidos, principalmente cangrejos, aunque se han reportado casos por la ingestión de carne cruda de jabalíes en Japón ^8^, o por contacto con manos y utensilios de cocina contaminados con las metacercarias -forma infecciosa del parásito- durante la preparación de estos crustáceos ^9^.

En Ecuador, se presenta en cualquier grupo de edad que ingiera las metacercarias, pero, generalmente, los niños son los más afectados por la costumbre de pescar en los riachuelos e ingerir en el sitio los crustáceos crudos, ahumados o asados. Los cangrejos de río tienen nombres populares como “apangoras” en la costa o “aparungas”, entre los kichwas de la amazonia. En las poblaciones rurales existe la creencia de que los cangrejos recién capturados en los riachuelos son “frescos” y buenos para el «chuchaqui» (resaca), y también, que aumentan la leche materna cuando son ingeridos crudos o en ceviche. En la amazonia, los cangrejos *Trichodactylus faxoni* y *Zilchiopsis ecuadoriensis* resultaron positivos para *P. mexicanus*^3^^,^^6^.

La paragonimiasis pulmonar es la forma clínica predominante y se caracteriza por síntomas como tos, dolor torácico posterior, expectoración con esputo herrumbroso y, a veces, franca hemoptisis; en las infecciones crónicas puede complicarse con derrame pleural, que requiere la hospitalización del paciente para su manejo ^10^^,^^11^. El diagnóstico errado de tuberculosis pulmonar es común porque las zonas endémicas para paragonimiasis también lo son para la tuberculosis pulmonar. Aproximadamente, el 12,9 % de los casos de paragonimasis fueron diagnosticados y tratados como tuberculosis en una zona costera de Ecuador ^3^. Según el portal web de egresos hospitalarios del Ministerio de Salud Pública de Ecuador, entre el 2021 y el 2023 se hospitalizaron seis pacientes (3 hombres y 3 mujeres), registrados en la provincia amazónica Morona Santiago y las regiones subtropicales de las provincias de Pichincha y Santo Domingo de los Tsáchilas ^12^.

El método diagnóstico de referencia es la observación microscópica de los huevos operculados en el esputo. Los exámenes imagenológicos son complementarios en el diagnóstico, aunque no son específicos. La radiografía de tórax y las tomografías computarizadas pueden revelar infiltrado difuso, nódulos, lesiones semejantes a sombras anulares, cavidades, opacidades lineales, abscesos pulmonares, derrame pleural y neumotórax ^11^. Más del 50 % de los pacientes hospitalizados presentaron leucocitosis (11.000 a 15.000 células/μΙ) con hipereosinofilia, mientras que unos pocos tenían valores bajos de hemoglobina y hematocrito ^13^.

El tratamiento farmacológico de elección recomendado por la OMS es el triclabendazol oral (10 mg/kg en una o dos dosis diarias durante uno o dos días), porque los pacientes toleran mejor el medicamento cuando se administra en dosis única; como alternativa, está el praziquantel oral diario (75 mg/kg durante tres días) ^1^^,^^7^. El Ministerio de Salud Pública de Ecuador incluye al praziquantel en su “Cuadro nacional de medicamentos básicos y registro terapéutico nacional” ^14^. Los dos fármacos tienen una eficacia casi del 100 %, con pocos y pasajeros efectos adversos.

Se presentan el diagnóstico y el tratamiento con triclabendazol de la paragonimiasis pulmonar en un niño indígena kichwa remitido a Quito desde la amazonia, con sintomatología pulmonar de cuatro años de evolución.

## Presentación del caso

**Figura 1:**
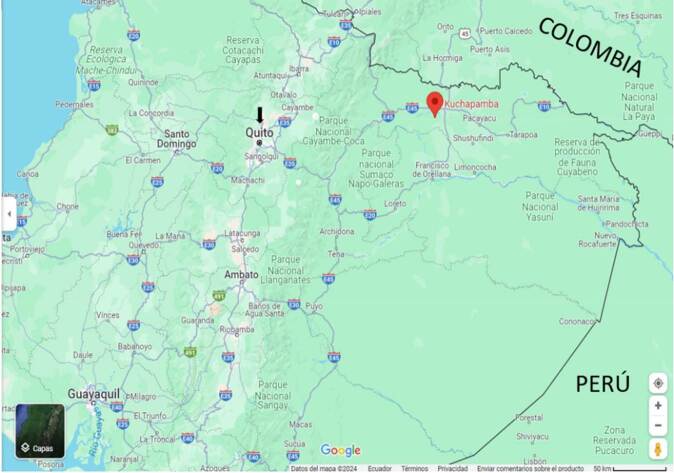
Mapa de Ecuador. La comunidad rural de Kuchapamba (símbolo rojo) donde el niño nació, reside y refirió la ingestión de cangrejos de río, está ubicada al norte de la selva amazónica ecuatoriana, a 60,4 km de la frontera con Colombia. La ciudad de Quito (flecha negra), capital del Ecuador, donde se diagnosticó y trató la paragonimiasis pulmonar, está ubicada en la región andina.

Se trata de un niño indígena de 10 años perteneciente al pueblo kichwa, nacido y residente en la comunidad Kuchapamba (0° 03’ 20,1” N; 76° 58’ 29,5” O) de la parroquia Santa Cecilia, Cantón Lago Agrio, provincia de Sucumbíos, al norte de la selva amazónica ecuatoriana (figura 1).

Fuente: Google Maps, s.f.

El niño fue remitido desde el Hospital General Marco Vinicio Iza, ubicado en Nueva Loja (a 35,4 km de Kuchapamba), por presentar síntomas respiratorios como tos y expectoración herrumbrosa y, a veces, franca hemoptisis, desde hacía cuatro años. Su padre refirió que ocasionalmente presentaba fiebre y que había sido hospitalizado en tres ocasiones; los diagnósticos presuntivos fueron tuberculosis pulmonar y neumonías. El abuelo paterno falleció por tuberculosis pulmonar. No obstante, todos los exámenes de esputo del paciente fueron negativos para *Mycobacterium tuberculosis*. En la última hospitalización, fue necesario colocarle un tubo de tórax para drenar el derrame pleural.

Ante la recurrencia y persistencia de los síntomas mencionados, y al no tener un diagnóstico etiológico, el paciente fue remitido al Hospital Pediátrico Baca Ortiz de Quito (ubicado en la región andina, a 293 km de Nueva Loja), para investigar la causa de los síntomas pulmonares. El padre manifestó que la dieta familiar incluía la ingestión de cangrejos de agua dulce recolectados en los riachuelos cercanos. Además, ingieren peces, moluscos y carne de animales silvestres como jabalíes y guantas (*Cuniculus* spp.), entre otros.

Durante el examen de ingreso al hospital en Quito, el paciente presentaba temperatura de 36 °C, tensión arterial de 115/76 mm Hg, frecuencia cardiaca de 60 latidos por minuto y frecuencia respiratoria de 24 respiraciones por minuto. Pesaba 26,8 kg, con talla de 126 cm y tenía un índice de masa corporal de 16,9 kg/m^2^. Se encontraba consciente, orientado en espacio, tiempo y persona, con un puntaje en la escala de Glasgow de 15/15 y sin signos neurológicos de focalización. No se palparon adenopatías. Su tórax era simétrico, con conservación de la capacidad de expansión y disminución del murmullo vesicular en la base pulmonar izquierda, y sin ruidos sobreagregados ni dificultad respiratoria. Los demás hallazgos del examen físico fueron normales.

En los primeros días de hospitalización, el paciente expectoró abundante esputo herrumbroso, con estrías de sangre, especialmente en las mañanas (figura 2A). Estas secreciones se recolectaron para el examen microscópico en búsqueda de huevos de *Paragonimus* spp., bacilos de *M*. *tuberculosis* y levaduras.

**Figura 2A:**
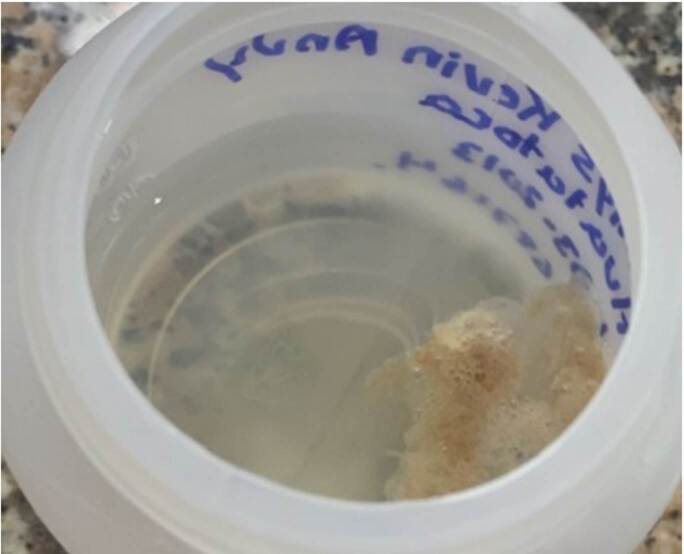
Muestra de esputo de aspecto mucoide, coloración herrumbrosa (color ladrillo) y estrías o vetas sanguinolentas, en la cual se observaron al microscopio abundantes huevos operculados característicos de *Paragonimus* spp.

La biometría hemática reportó 6,1 x 10^3^ leucocitos/μl (rango de referencia: 4,4 - 11,0 x 10^3^células/μl); 5,0 x 10^3^neutrófilos/μl (2,5 - 7,5); 3,1 x 10^3^ linfocitos/μl (3,0 - 9,5) y 2,24 x 10^3^ eosinófilos/μl (1,0 - 1,5); hemoglobina de 9,7 g/dl (9,5 - 13,0), hematocrito de 29,6 % (30 - 44 %) y 306.000 plaquetas/μl (150.000 - 450.000).

Además, los resultados incluyeron: glucosa de 102 mg/dl (100 - 180); urea de 6,8 mg/dl (5 - 18); creatinina de 0,17 mg/dl (0,3 - 0,7); transaminasa glutámico-oxalacética de 25,6 U/L (0 - 37); transaminasa glutámico-pirúvica de 28,6 U/L (> 60); γ-glutamil-transferasa de 22 U/L (11 - 50); bilirrubina total de 0,10 mg/dl (1,0); bilirrubina directa de 0,04 mg/dl (0,3); fosfatasa alcalina de 179 U/L (hasta 350); lactato deshidrogenasa de 203 U/L (170 - 580); proteínas totales de 4,5 g/dl (4,4 - 5,4); albúmina de 6,88 g/dl (6,2 - 8,0) y proteína C reactiva de 1,33 mg/L (hasta 10). El examen de heces no reveló quistes ni huevos de parásitos.

En el estudio microscópico de esputo en fresco (sin coloración), se observaron abundantes huevos, operculados y ovoides, indicativos de *Paragonimus* spp. (figura 2B). Con la coloración de Ziehl-Neelsen y ácido peryódico de Schiff, no se observaron bacilos ni esporas de hongos.

**Figura 2B:**
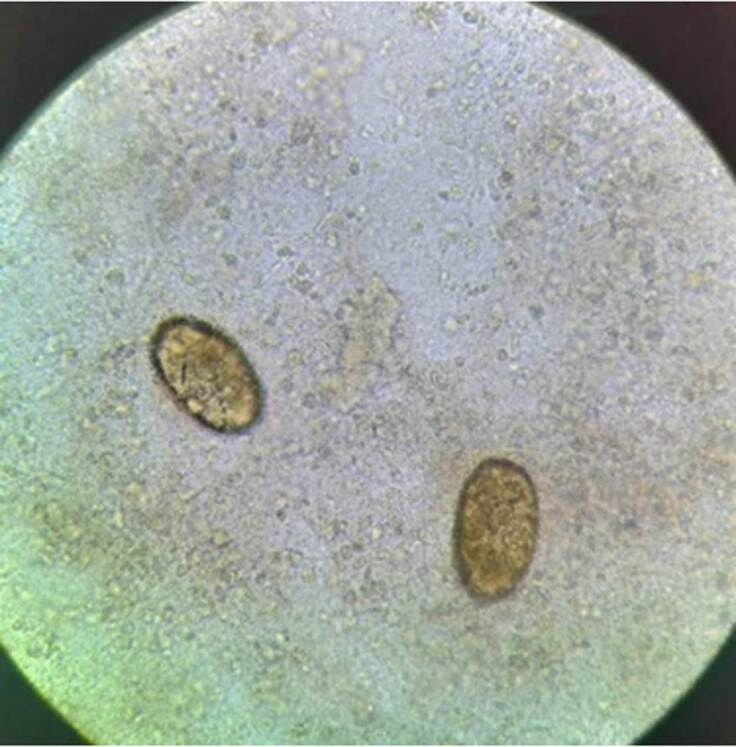
Muestra de esputo, 40X. Se observaron huevos ovoides de 80 a 110 μm por 50 a 70 μm, de color marrón amarillento, ovoides, con pared gruesa. Uno de los extremos está ligeramente aplanado y es donde se encuentra el opérculo.

**Figura 3:**
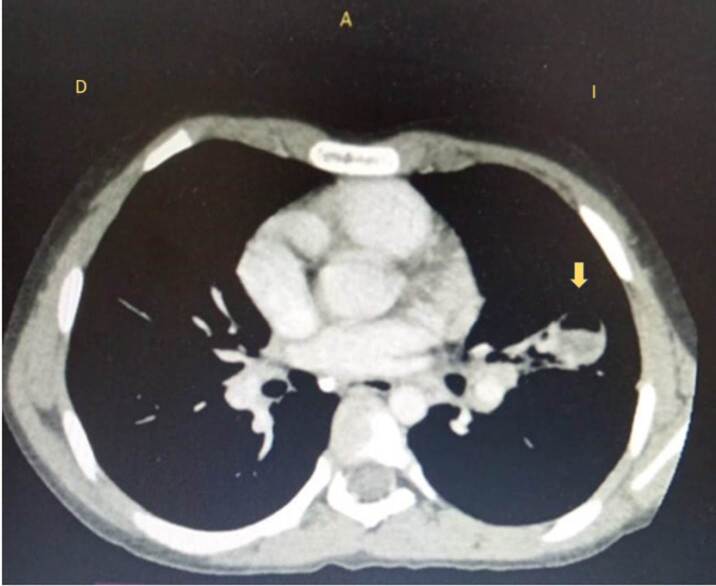
Tomografía axial computarizada de tórax al ingreso al hospital pediátrico en Quito. La flecha amarilla indica la lesión redondeada con nivel hidroaéreo en su interior descrita en el reporte.

En la tomografía axial computarizada de tórax, simple y con contraste, se observó en la língula una lesión redondeada de 22 x 20 x 33 mm y de un volumen aproximado de 8 ml con nivel hidroaéreo y focos de gas en la parte anterior, además de áreas consolidadas alrededor de dicha lesión. En el campo basal izquierdo, se visualizó una imagen con patrón de vidrio esmerilado (figura 3).

Hecho el diagnóstico parasitológico de paragonimiasis pulmonar, se solicitó el envío de praziquantel o triclabendazol al Ministerio de Salud Pública de Ecuador, y respondieron no disponer de ninguno de los dos fármacos. Se consiguió triclabendazol en tabletas de 250 mg de Egaten^®^ (Novartis) por medio de la organización no gubernamental “Centro de Epidemiología Comunitaria y Medicina Tropical”, en Esmeraldas, y se administró por vía oral una dosis única diaria de 10 mg/kg durante dos días. Luego de cinco días de hospitalización, desaparecieron los síntomas pulmonares. El paciente no presentó efectos adversos ni secundarios, por lo cual fue dado de alta.

En el último control, realizado un año después de terminar el tratamiento, el padre informó que el paciente había presentado tos esporádica sin expectoración herrumbrosa. El paciente fue sometido a un examen físico cuyos hallazgos fueron normales, y la microscopia de esputo para huevos de *Paragonimus* spp. y bacilos de *M*. *tuberculosis* fue negativa.

## Consideraciones éticas

Se obtuvo el consentimiento informado firmado por el padre del niño, para la publicación de este caso con fines académicos.

## Discusión

El reporte de este caso de paragonimiasis pulmonar es significativo pues fue diagnosticado y tratado en un hospital de primer nivel ubicado en la región andina, cuando podría haberse diagnosticado en el hospital de su localidad amazónica, desde donde fue remitido.

Esto tiene implicaciones negativas en la salud pública. Todos los médicos y laboratoristas deberían estar entrenados para diagnosticar y tratar oportunamente estos casos de enfermedades tropicales, pero no es así; esta es la razón para considerar a la paragonimiasis como una de las 20 enfermedades tropicales olvidadas o desatendidas ^1^.

Si bien su diagnóstico clínico es inespecífico y puede confundirse con otras enfermedades pulmonares infecciosas, como la tuberculosis o la histoplasmosis, el diagnóstico por el laboratorio es simple, rápido, específico y barato; consiste en la observación de una muestra de esputo en fresco al microscopio con un aumento de 10X o 40X.

La suspicacia diagnóstica de los médicos debe orientarse a las enfermedades endémicas y de importancia epidemiológica en las regiones tropicales. El conocimiento de que la paragonimiasis pulmonar es endémica en las regiones tropicales y subtropicales de la costa y la amazonia ^3^^,^^12^, facilita su diagnóstico diferencial. Por esto, es importante indagar sobre antecedentes de ingestión de crustáceos de agua dulce por parte de los pacientes o de sus familiares. En este caso, el padre refirió la ingestión de “aparungas” en la dieta familiar.

Los métodos diagnósticos de imagenología (radiografía, tomografía axial computarizada y resonancia magnética) de tórax, no son específicos, ya que en las imágenes se observan nódulos, cavitaciones, abscesos pulmonares y hasta derrames pleurales, que pueden corresponder a otras causas, como tuberculosis, neumonía lobar, cáncer, etc. ^15^. El examen de sangre es útil por la eosinofilia que se presenta en la mayoría de los pacientes ^16^, como sucedió en este caso.

En este reporte, se diagnosticó paragonimiasis en un niño indígena kichwa, nacido y residente en el norte de la selva amazónica, donde la biodiversidad es abundante y la existencia de cangrejos infectados con metacercarias de *P*. *mexicanus* ha sido demostrada ^3^^,^^6^. Los indígenas amazónicos tienen la costumbre de consumir crustáceos crudos, asados o ahumados como parte de la dieta familiar; incluso, la cultura gastronómica o las creencias de que los cangrejos son “frescos” y aumentan la leche materna, incrementan el riesgo de contagio ^3^. Dada la posibilidad de que existan más infectados en las comunidades aledañas de los países vecinos, Colombia y Perú, se sugiere realizar estudios de búsqueda activa; en reportes anteriores, se demostraron importantes prevalencias en la amazonia y la costa ^4^^,^^7^.

En el presente caso, la ingestión de cangrejos de agua dulce fue la vía más probable de infección. Sin embargo, se debería investigar la posibilidad de que los jabalíes -usualmente cazados y comidos por los indígenas amazónicos- puedan estar infectados. Se conoce de casos en Asia cuya fuente de infección fue la ingestión de carne de jabalíes ^8^.

No se llegó a determinar la especie de *Paragonimus*, ya que para ello se requerían métodos moleculares que no estaban disponibles en ese momento. Sin embargo, es posible que se trate de *P*. *mexicanus*, la especie reportada para Centroamérica y Suramérica, incluido Ecuador ^3^^,^^10^.

La falta de diagnóstico y de tratamiento oportuno conlleva la cronicidad de la enfermedad y complicaciones como derrame pleural, pérdida de peso y anemia ^10^^,^^16^. El niño presentaba tos con expectoración herrumbrosa desde cuatro años antes, con varias hospitalizaciones previas. En la última hospitalización, presentó derrame pleural, probablemente causado por la misma paragonimiasis, pero los diagnósticos fueron neumonía o tuberculosis. Es común confundir esta enfermedad con la tuberculosis y errar en su tratamiento ^3^, porque la paragonimiasis también presenta complicaciones como derrame pleural y anemia ^10^^,^^16^.

En el líquido pleural se pueden encontrar huevos de *Paragonimus* spp., aunque no siempre se evidencian. Sin embargo, se debe solicitar su búsqueda en aquellos pacientes con derrame pleural, provenientes de zonas tropicales endémicas de paragonimiasis. Según el Ministerio de Salud Pública de Ecuador, seis casos fueron hospitalizados en los últimos dos años ^12^. El paciente de este reporte presentaba disminución del hematocrito (29,6 %; rango normal: 30 - 44 %) y una hemoglobina de 9,7 g/ dl (rango normal: 9,5 - 13,0), lo que sugiere una probable anemia debido a la hemoptisis asociada con la paragonimiasis.

En este caso, la administración de triclabendazol (dosis única diaria por dos días) fue eficaz y no causó efectos adversos. Según la OMS y Calvopiña *et al*. (2003), este es el tratamiento preferido por consistir en la administración de una dosis única diaria y no de tres al día, como ocurre con el praziquantel ^1^^,^^7^. Se consiguió el triclabendazol por medio de una organización no gubernamental porque el Ministerio de Salud Pública de Ecuador no dispone de ninguno de los dos fármacos mencionados. Se le sugiere al Ministerio adquirir los dos fármacos por medio de la OMS ^17^. Es importante mencionar que el triclabendazol también es el fármaco de primera línea para el tratamiento de la fascioliasis y que el praziquantel es útil para otras trematodiasis y cestodiasis, como las teniasis intestinales y la neurocisticercosis.

En conclusión, este caso hace evidente la falta de un diagnóstico oportuno, tanto por clínica como por laboratorio, de la paragonimiasis pulmonar en las zonas endémicas de la región amazónica. Esta deficiencia puede llevar a la cronicidad de la enfermedad, el desarrollo de complicaciones, el uso indebido de medicamentos y hospitalizaciones innecesarias. Por lo tanto, es fundamental capacitar a médicos y laboratoristas en el adecuado abordaje diagnóstico de esta enfermedad, para evitar el sufrimiento del paciente y sus familiares, así como la pérdida de tiempo y recursos de las familias y del gobierno.

Además, se recomienda promover la educación en salud entre los habitantes de las comunidades endémicas, enfatizando sobre la importancia de consumir bien cocinados los crustáceos de agua dulce.
